# Supplementary material for: Generation and Characterization of a New Preclinical Mouse Model of EGFR-Driven Lung Cancer with MET-Induced Osimertinib Resistance
Source: Cancers (Basel). 2021 Jul 9;13(14):3441. doi: 10.3390/cancers13143441 (PMC8307933; doi:10.3390/cancers13143441)
Supplement: Supplementary file 1 [file cancers-13-03441-s001.zip › Figure S1-S4.pdf]

Generation and Characterization of a New Preclinical Mouse Model of EGFR-Driven Lung Cancer with MET-Induced Osimertinib Resistance

Maicol Mancini, Quentin-Dominique Thomas, Sylvia Bourdel, Laura Papon [1], Emilie Bousquet, Prisca Jalta, Silvia La Monica, Camille Travert, Roberta Alfieri, Xavier Quantin, Marta Cañamero and Antonio Maraver

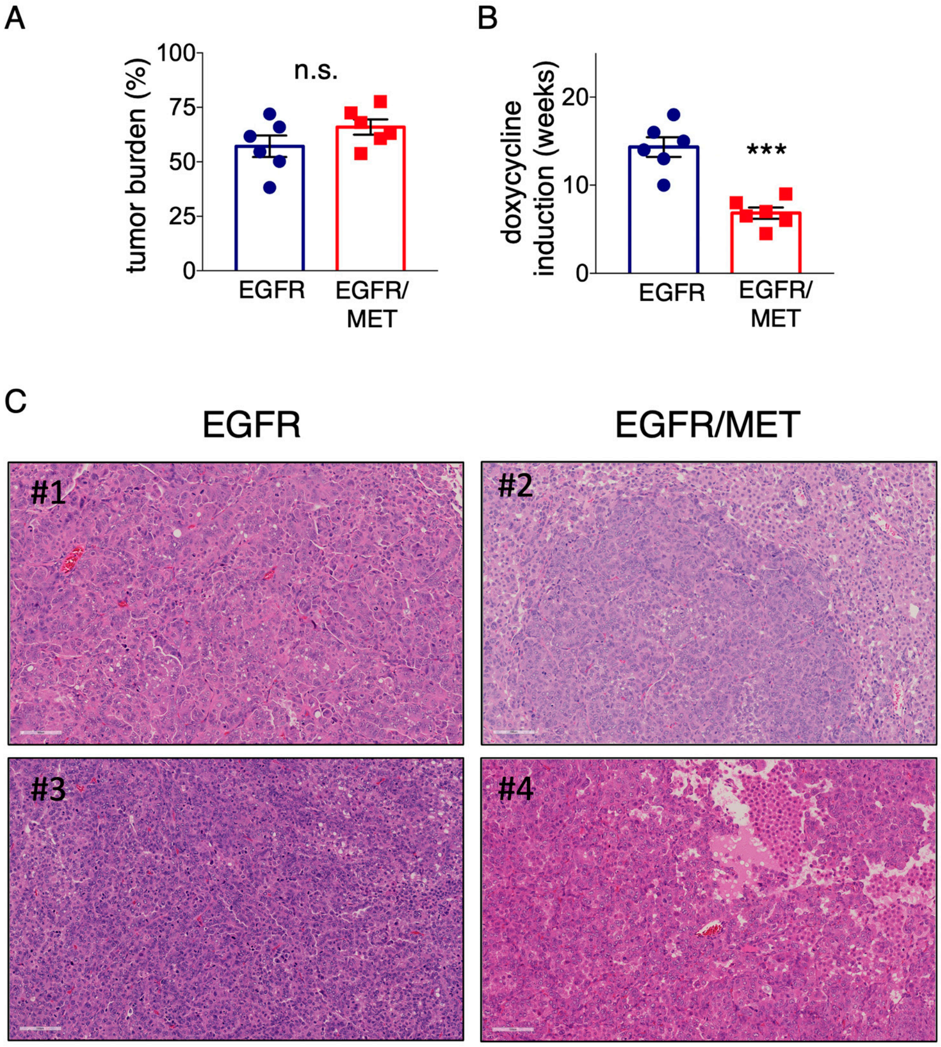

Figure S1. (**A**) Tumor burden in hematoxylin & eosin-stained lung samples from EGFR (n = 6) and EGFR/MET (n = 6) mice (see Figure 1C). Data are the mean ± SEM; n.s., not significant (unpaired *t*-test). (**B**) Survival time after MET and EGFR transgene induction with doxycycline (same mice as in A). Data are the mean ± SEM; *** $p \leq 0.001$ (unpaired *t*-test). (**C**) Four examples of adenocarcinoma in EGFR (mice 1 and 3) and EGFR/MET (mice 2 and 4) mice. All were solid adenocarcinomas with high proliferation rate, as indicated by the several metaphase mitotic figures in metaphase. In mouse 2, the tumor was surrounded by a dense infiltrate with macrophages and plasma cells. In mouse 4, the presence of edema with macrophages could be observed. Scale bar, 100 µm.

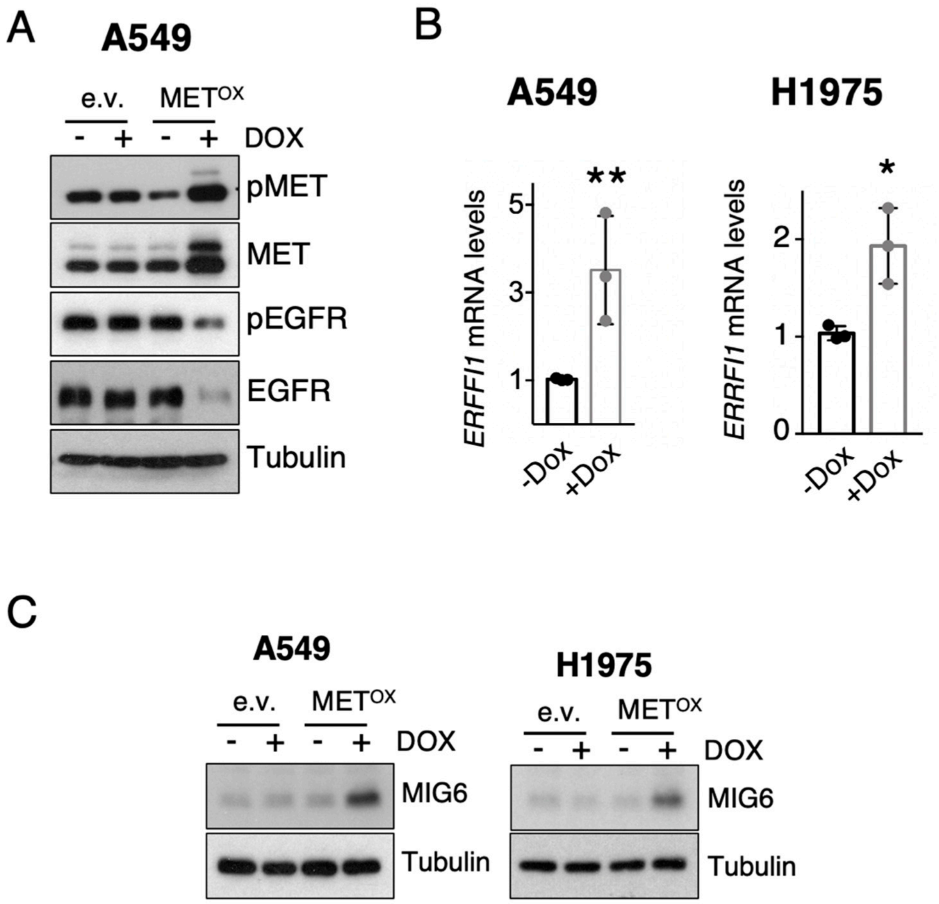

Figure S2. (**A**) Immunoblotting of the indicated proteins in A549 cells infected with lentiviral particles harboring the doxycycline-inducible MET construct (MET^{OX}) or empty vector (e.v.). Cells were incubated (+) or not (-) with 1 µg/mL of doxycycline for 48 h. This is a representative example of an experiment performed twice. (**B**) *ERRFI1* mRNA levels in A549 and H1975 cells infected with lentiviral particles harboring the doxycycline-inducible MET construct (MET^{OX}, $n = 3$). Cells were treated or not with 1 µg/mL of doxycycline for 48 h. Values are the mean ± SD; * $p \leq 0.05$, ** $p \leq 0.01$ (unpaired *t*-test). (**C**) Immunoblotting of the indicated proteins in A549 and H1975 cells treated as in A. This is a representative example of an experiment performed three times.

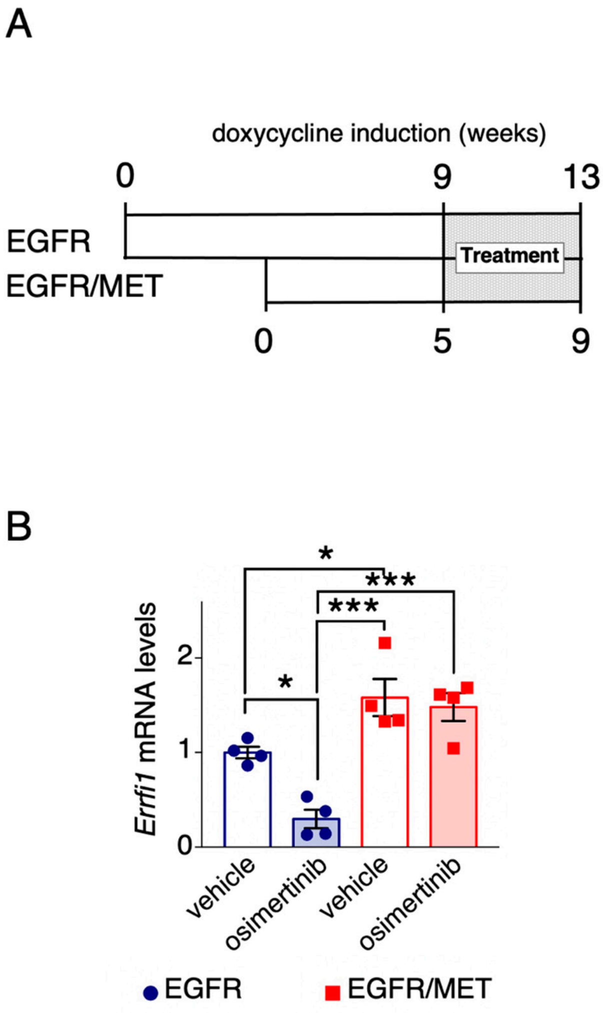

Figure S3. (**A**) The cartoon depicts the experimental setting of Figure 3. (**B**) *Errfi1* mRNA expression levels in lung tumors from EGFR and EGFR/MET mice treated with vehicle or osimertinib (n = 4 tumors/group). Values are the mean ± SEM; *p ≤ 0.05, ***p ≤ 0.001 (one-way ANOVA followed by the Tukey post-hoc test).

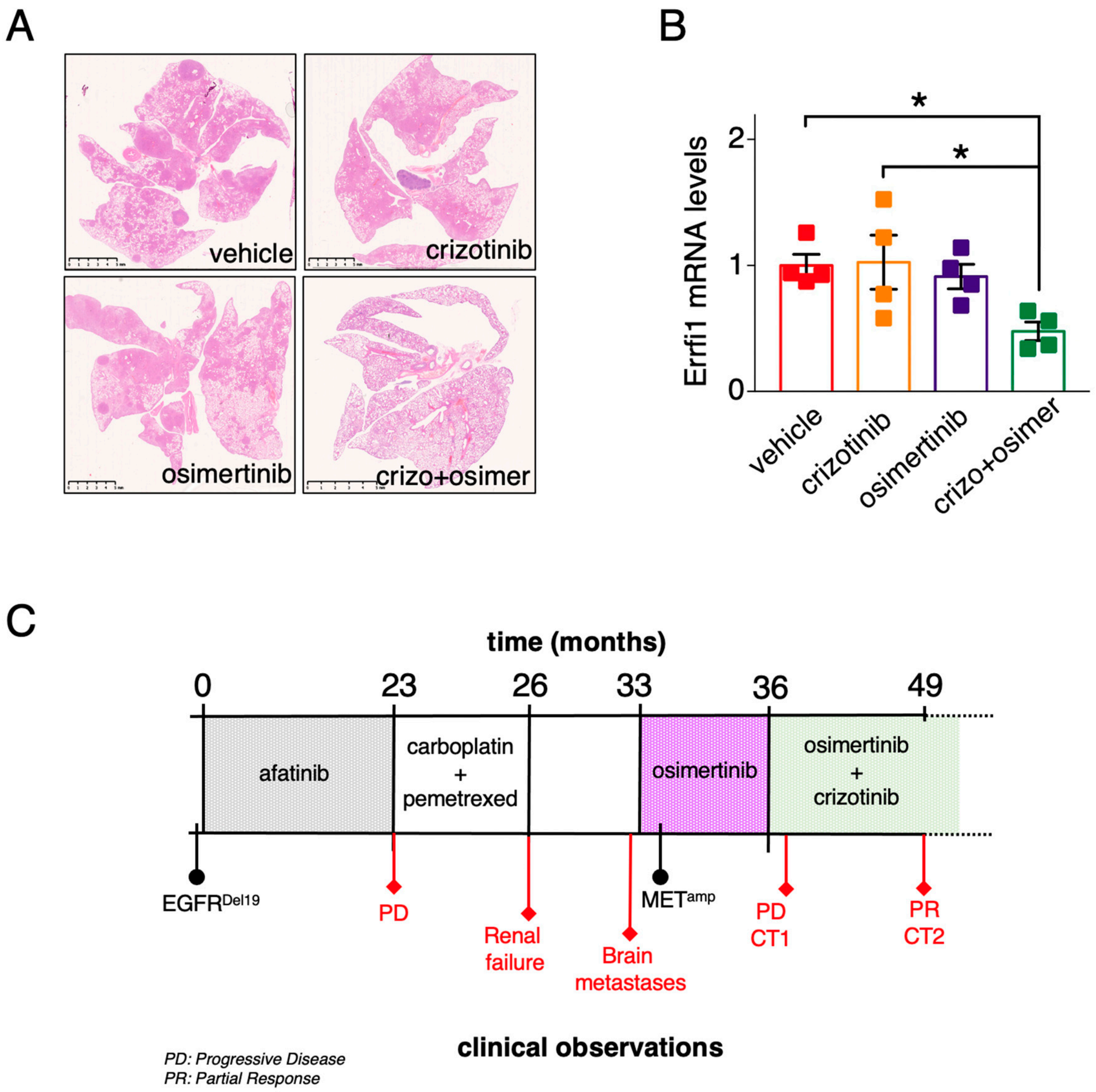

Figure S4. (**A**) Examples of hematoxylin and eosin staining of whole lungs from EGFR/MET mice treated as described in A. Scale bar, 5 mm. (**B**). *Errfi1* mRNA expression levels in lungs from EGFR/MET mice treated as described in A ($n = 4$ animals per treatment group). Values are mean ± SEM; * $p \leq 0.05$ (one-way ANOVA followed by the Tukey post-hoc test). (**C**) The cartoon depicts the clinical observation and treatments of a case report referring to Figure 4E.
