# Supplementary material for: Generation and Characterization of a New Preclinical Mouse Model of EGFR-Driven Lung Cancer with MET-Induced Osimertinib Resistance
Source: Cancers (Basel). 2021 Jul 9;13(14):3441. doi: 10.3390/cancers13143441 (PMC8307933; doi:10.3390/cancers13143441)
Supplement: Supplementary file 1 [file cancers-13-03441-s001.zip › Figure S5WB.pdf]

Figure 1C

EGFR				EGFR/MET			
1	2	3	4	5	6	7	8

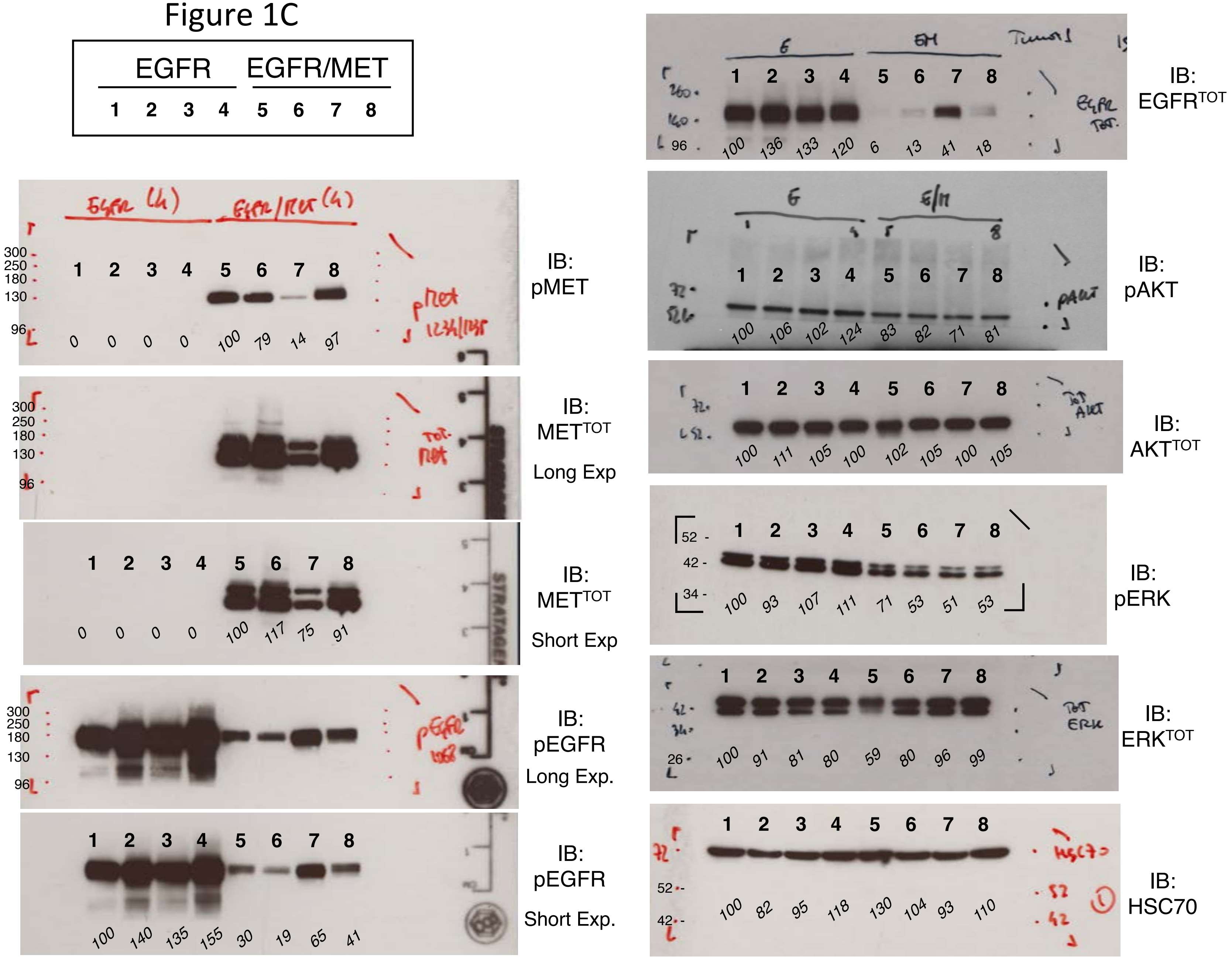

Figure 2B

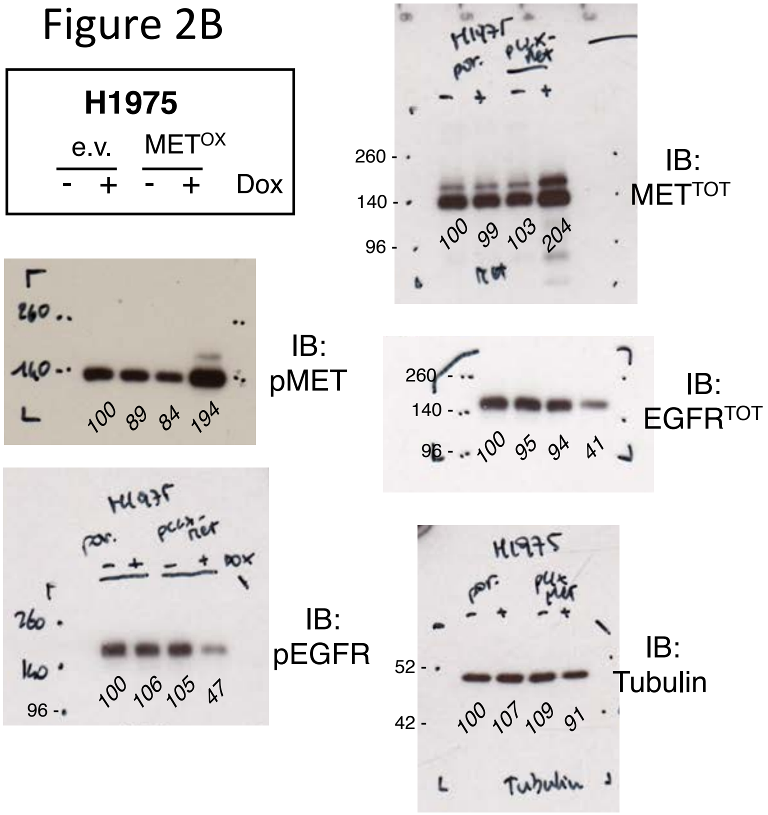

Suplemental Fig2A

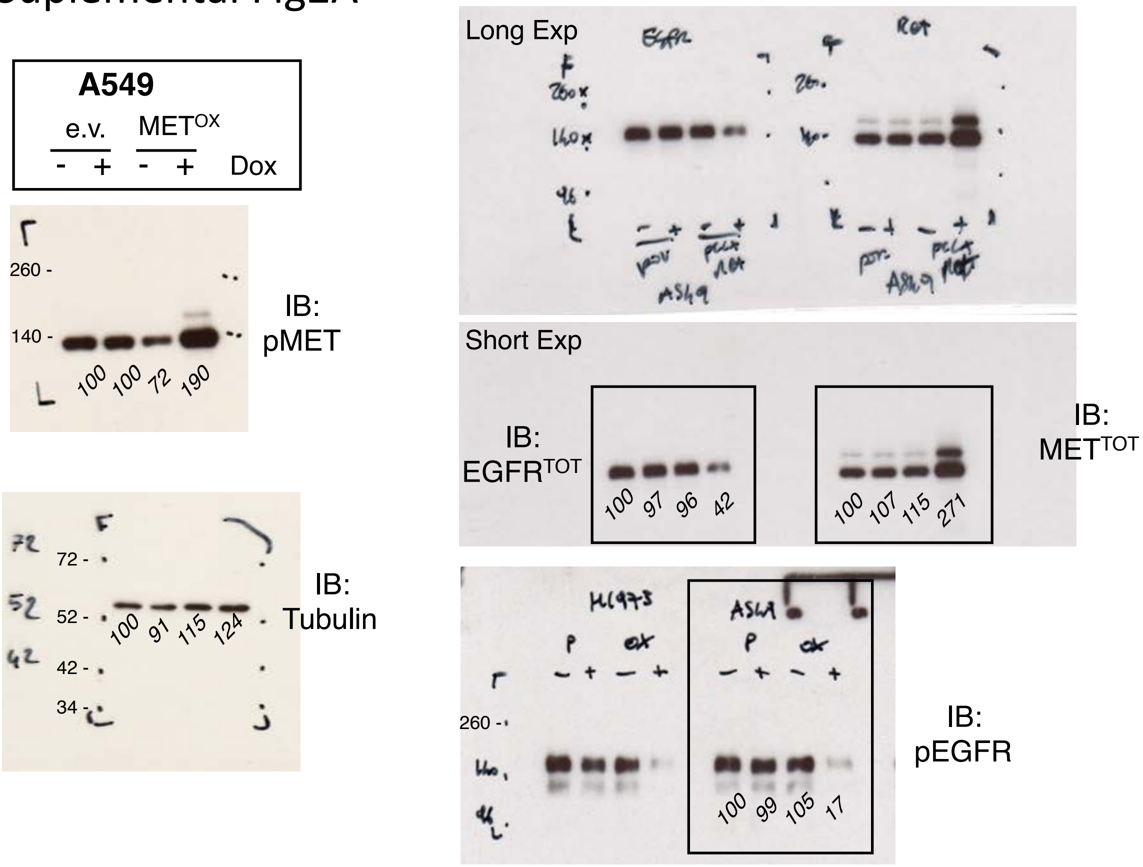

Figure 3C

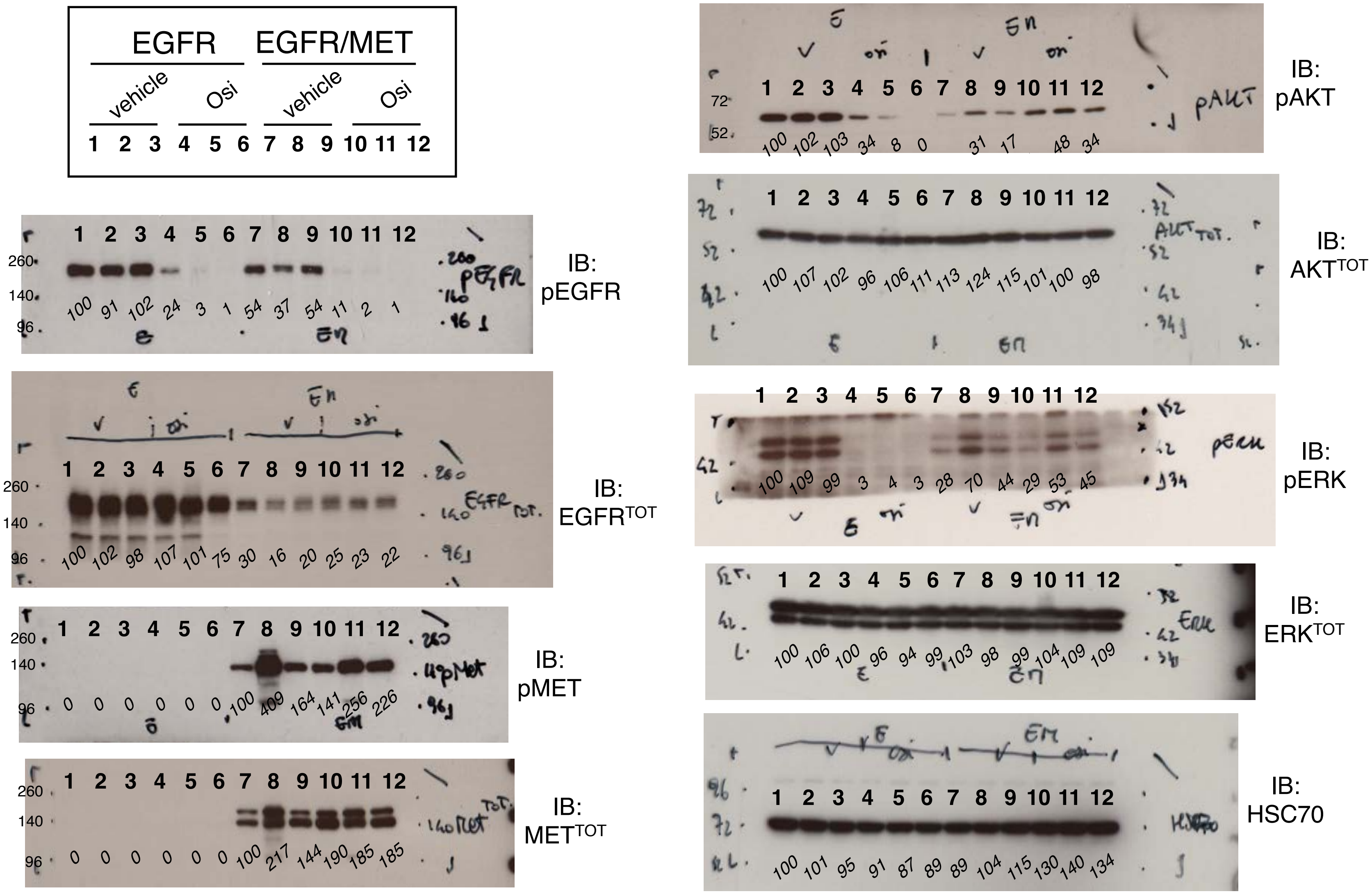
EGFR
EGFR/MET
vehicle
Osi
vehicle
Osi
1 2 3 4 5 6 7 8 9 10 11 12
IB: pEGFR
IB: $EGFR^{TOT}$
IB: pMET
IB: MET^{TOT}
IB: pAKT
IB: AKT^{TOT}
IB: pERK
IB: ERK^{TOT}
IB: HSC70

Figure 4D

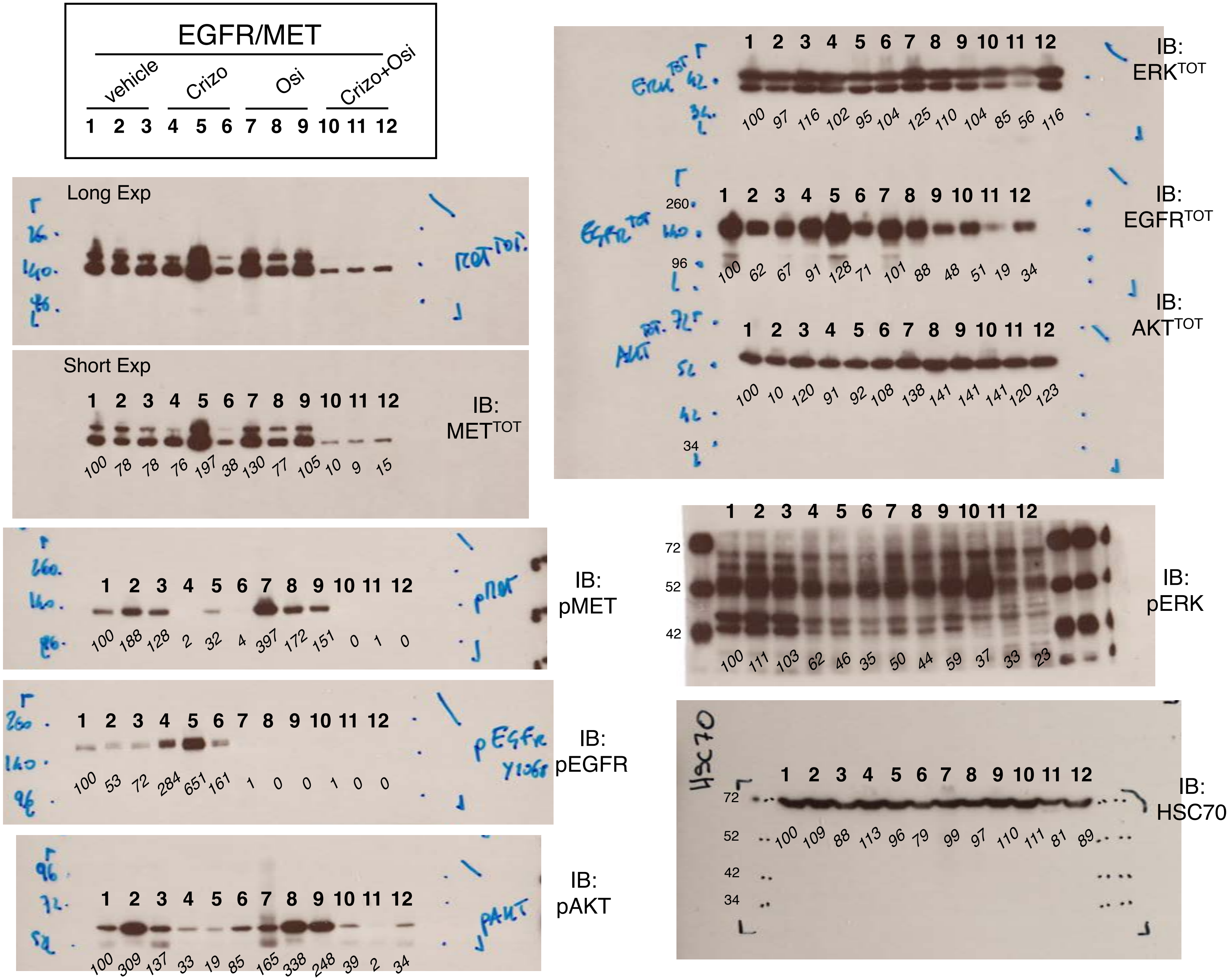
EGFR/MET
vehicle
Crizo
Osi
Crizo+Osi
1 2 3 4 5 6 7 8 9 10 11 12
Long Exp
Short Exp
IB: MET^TOT
100 78 78 76 197 38 130 77 105 10 9 15
IB: pMET
100 188 128 2 32 4 397 172 151 0 1 0
IB: pEGFR
100 53 72 284 651 161 1 0 0 1 0 0
IB: pAKT
100 309 137 33 19 85 165 338 248 39 2 34
IB: ERK^TOT
100 97 116 102 95 104 125 110 104 85 56 116
IB: EGFR^TOT
260
96
100 62 67 91 128 71 101 88 48 51 19 34
IB: AKT^TOT
34
100 10 120 91 92 108 138 141 141 141 120 123
IB: pERK
72
52
42
100 111 103 62 46 35 50 44 59 37 33 23
IB: HSC70
72
52
42
34
100 109 88 113 96 79 99 97 110 111 81 89
